# Indications and Yield of Pediatric Endoscopy in Bahrain: A Tertiary Center Experience

**DOI:** 10.1155/2022/6836842

**Published:** 2022-03-26

**Authors:** Hasan M. A. Isa, Fatema Alfayez

**Affiliations:** ^1^Pediatric Department, Arabian Gulf University, Manama, Bahrain; ^2^Pediatric Department, Salmaniya Medical Complex, Manama, Bahrain

## Abstract

**Results:**

Of 1,111 patients, 1,101 (99.1%) were included in the study. 589 (53.6%) patients were males. Median age at the time of endoscopy was 8 (interquartile range 3, 11) years. 1534 endoscopies were performed (1193 upper GI endoscopies (UGIE) and 341 colonoscopies) in 1296 sessions. The mean number of endoscopies per year was 59 ± 30.9 procedures with 81.4% reduction noted after coronavirus pandemic (*P* < 0.0001). Ratio between UGIE to colonoscopy was 3.5 : 1. Median number of endoscopies per patient was one, ranging from one to eight procedures. 1153 (89%) sessions were diagnostic, and 143 (11.0%) were therapeutic. Main endoscopic indication was chronic abdominal pain (451 (40.9%) patients) followed by upper GI bleeding (302 (27.4%) patients). Overall positive yield was 68.1% (716/1052 procedures). Endoscopic yield varies according to the type of procedure (*P* = 0.003). Colonoscopy alone gave a higher yield (82.6%, 38/46 procedures) compared to combined procedures (75.4%, 141/187) and UGIE alone (65.6%, 537/819).

**Conclusions:**

This study emphasizes a careful selection of the type of endoscopic procedures, based on the expected endoscopic yield, to diagnose and treat pediatric GI diseases. In patients with chronic abdominal pain, endoscopy should be reserved as a second-line tool to avoid unnecessary use of invasive procedures.

## 1. Introduction

Gastrointestinal (GI) endoscopy in children is a field that has been evolving in the last decades [1]. Innovative advance in pediatric endoscopy has led to a more accurate diagnosis of common and new GI diseases [2, 3]. Yet, in developing countries, upper gastrointestinal endoscopy (UGIE) is an underutilized method in the management of pediatric disorders [4]. However, pediatric UGIE has been performed as twice as colonoscopy [5]. Colonoscopy is also a valuable procedure for children with lower gastrointestinal (LGI) diseases [6]. Pediatric colonoscopy was introduced slightly later than UGIE, as the first reports of colonoscopy in children appeared in the late 1970s [6]. In certain circumstances, both upper and lower GI endoscopy are required to be performed during the same session, as seen in patients with chronic diarrhea [1]. Pediatric endoscopy has been proven to be a safe and effective diagnostic and therapeutic tool for pediatric GI diseases [1, 2, 6–8]. Currently, it has been used to evaluate children of all ages including infants [1, 2, 8].

Diagnostic indications of pediatric endoscopy are generally different from those of adults as screening for cancer is extremely low in the pediatric age groups [9]. On the other hand, chronic abdominal pain (CAP) is one of the most common indications of UGIE in children [1, 2, 8, 10, 11]. Therapeutic indications such as esophageal dilatation and foreign body removal are also important [2, 12–15].

Studying the yield of endoscopic procedures is essential. GI endoscopy is usually associated with positive findings [2, 5]. Positive endoscopic yield is higher in patients with alarming red flag signs compared to those without [8].

The situation of pediatric endoscopy in Europe and worldwide is unclear [16]. Moreover, information on the utility of GI endoscopies in children is scarce as only few reports were available from developing countries [4, 8]. There is a need to raise the awareness of the diagnostic and therapeutic role of pediatric endoscopy in developing countries [4].

In Bahrain, there has been no published data about the indications and the yield of endoscopic procedures in children. This study aimed to assess the appropriateness of the indications of endoscopic procedures in children attended the main tertiary hospital in Bahrain, and to evaluate the diagnostic and therapeutic yield of the pediatric GI endoscopy, keeping in mind the existing limitations to access this facility.

## 2. Material and Methods

### 2.1. Study Design and Setting

In a retrospective, cross-sectional, analytical study, medical records of all children (from birth to 18 years) who underwent GI endoscopy in the pediatric department, Salmaniya Medical Complex (SMC), the kingdom of Bahrain, from 1995 to 2020 were reviewed. SMC is the only tertiary care hospital in Bahrain providing both diagnostic and therapeutic GI endoscopy services to pediatric populations from all over the country. Pediatric GI endoscopies are performed by three senior gastroenterologists. Two types of endoscopes are used: Olympus (PCF-230 and XQ-230) or Pentax endoscopes (EG-2901 and EC-380IF). In addition, other endoscopic equipments are also used for different endoscopic indications, such as basket forceps for foreign body removal, snare cauterization forceps for polyp removal, and Savary-Gilliard bougies and balloon dilators for esophageal stricture dilatation. The procedures are performed under general anesthesia.

### 2.2. Study Participants

All pediatric patients referred for GI endoscopy, from the outpatient clinics, inpatient wards, and accident and emergency departments during the study period, were enrolled in the study. Patients older than 18 years of age, those who underwent endoscopic procedures before the year 1995, and those missing important relevant data were excluded.

### 2.3. Data Collection

From 1995 to 2010, the data were retrieved from the archived medical records, as paper-based files, while to 2020, the data were retrieved from the I-Seha electronic medical records. Patients' demographic characteristics including age, sex, and nationality were obtained.

Indications of UGIE were classified based on North American Society for Pediatric Gastroenterology, Hepatology, and Nutrition (NASPGHAN) recommendations [17]. Indications for both UGIE and LGI endoscopies were also in parallel with the list of indications published in the literature [18, 19].

Endoscopic findings, intervention, yield, outcome, and any complications related to the procedure were also gathered. The positive yield of endoscopic procedures was defined by the presence of any endoscopic finding seen in the performed procedure including the need for therapeutic interventions. Negative yield was considered if no endoscopic finding was detected, or no intervention was required. The final diagnosis of each patient was reviewed.

### 2.4. Ethical Approval

All the endoscopic procedures were performed after a written consent from the patients' parent/guardian was obtained. This study was approved by the secondary care ethical subcommittee at SMC (IRB number: 135111020) on 11 of October 2020 and was conducted according to the principles of the Helsinki Declaration.

### 2.5. Statistical Analysis

Data were entered into Excel 2016 and then transferred to SPSS version 21 (IBM Corp. Released 2012. IBM SPSS Statistics for Windows, Version 21.0. Armonk, NY: IBM Corp) for analysis. Basic descriptive statistics were performed and displayed as frequency and percentage for categorical variables. Continuous variables were displayed as mean and standard deviation, if they were normally distributed, or as median and interquartile range, if they were not normally distributed. Patients were divided in groups of 5-year intervals. One-way ANOVA was used to compare changes in the frequencies and trend of pediatric procedures in different year periods (1995-2008, 2009-2011, 2012-2019, and 2020). Comparison between types of endoscopies in terms of endoscopic yield was carried out using Fisher's exact test. *P* values less than 0.05 were considered statistically significant.

## 3. Results

During the study period, 1111 patients underwent GI endoscopies. Ten patients were excluded because of the lack of enough data about the indication for endoscopy. Demographic data of the remaining 1101 (99.1%) patients who were included in the study are shown in Table 1.

Five hundred eighty-nine (53.6%) patients were males. Most patients were Bahraini, while 59 (5.4%) patients were non-Bahraini (20 patients were from India; nine from Pakistan; seven from Egypt; four each from Iraq and Bangladesh; three each from Syria, Saudi Arabia, the Philippines, and Yemen; two from Sudan; and one each from Palestine, Oman, Jordan, United States of America (USA), and China, and nine patients were nonspecific other nationalities). The most frequent age group was below five years (371 (34.1%) patients).

**Figure 1:**
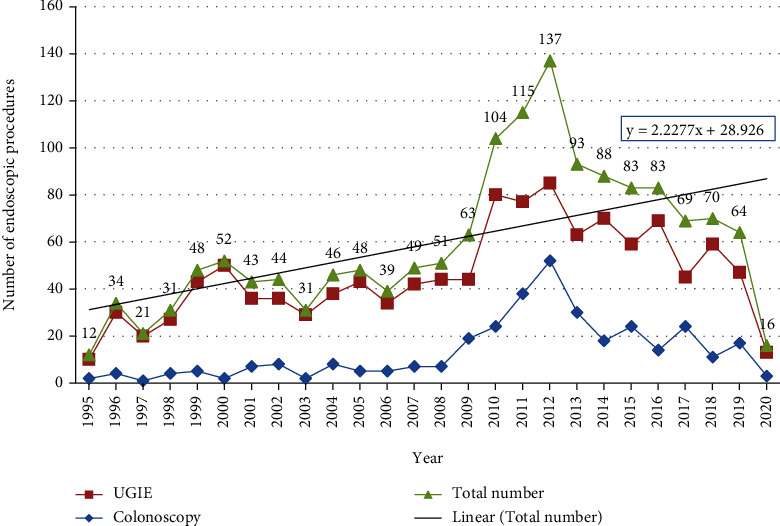
Number of gastrointestinal endoscopic procedures in children per year at Salmaniya Medical Complex, Bahrain (1995-2020), (*n* = 1534). UGIE: upper gastrointestinal endoscopy.

A total of 1534 endoscopic procedures were performed in 1296 sessions (955 for UGIE, 103 for colonoscopies, and 238 for combined procedures). The total number of UGI endoscopies was 1193 procedures, while the total colonoscopies were 341, with a ratio of 3.5 : 1. The number of GI endoscopic procedures per year is shown in Figure 1.

The overall mean number of endoscopic procedures per year was 59 ± 30.9 procedures. Between 1995 and 2008, the mean number of endoscopic procedures was 39 ± 12. There was a rapid peak in the number of procedures performed that started after the year of 2009 until 2011 (the mean was 94 ± 27) followed by a progressive reduction in the number of procedures noted more after the year of 2011 until 2019 (the mean was 86 ± 23), with a reduction rate of 8.5%. A further marked reduction was also noted in the year of 2020 (the mean was 16 ± 0), with a reduction rate of 81.4%. These differences were statistically significant (*P* < 0.0001).

The mean number of UGIE was 45.9 ± 19.8 endoscopies per year, while the median number of colonoscopies was 7.5 colonoscopies per year (IQR 4.0, 20.3).

**Figure 2:**
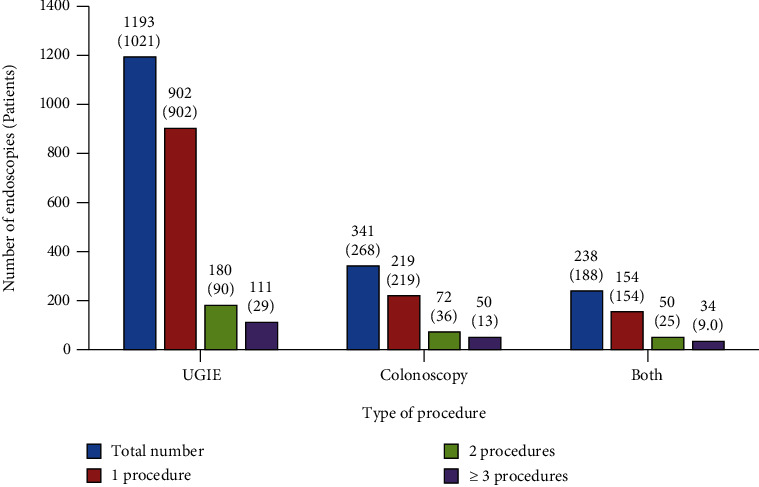
Number of pediatric gastrointestinal endoscopic procedures (*n* = 1534) per patient (*n* = 1101). UGIE: upper gastrointestinal endoscopy.

The total number of GI endoscopic procedures per patient is shown in Figure 2.

The median number of endoscopic procedures was one procedure per patient ranging from one to eight procedures.

Data about the indication of endoscopic procedures were available for all the patients (see Table 2).

Out of 1296 endoscopic sessions performed, 1153 (89%) sessions were diagnostic procedures. One hundred and forty-three (11.0%) therapeutic interventions were performed for 79 (7.2%) patients. Most of the interventions were for esophageal stricture dilatation, which was found in 27 (2.5%) patients who had 91 (63.6%) dilatation sessions. Forty-two (3.8%) patients had foreign body removal, five patients had removal or insertion of the feeding tube, and two patients had an injection of sclerotherapy for esophageal varices, while polypectomy, endoscopic retrograde cholangiopancreatography, and thermal cauterization for bleeding gastric ulcer secondary to *Helicobacter pylori*-induced gastritis were done for one patient each.

Data about the yield of endoscopic procedures were available for 1052 out of 1296 endoscopic sessions (81.2%) (Table 3).

The overall endoscopic procedures gave a positive yield of 68.1% (716/1052 procedures). There was a significant difference in the yield according to the type of procedure performed (*P* = 0.003). Colonoscopy alone gave a higher positive yield (82.6%) compared to combined procedures (75.4%) and UGIE alone (65.6%).

Out of 1,101 patients, data about the final diagnosis was available for 941 (85.4%) patients who had a total of 1084 final diagnoses; 233 of them had more than one final diagnosis. For the remaining 160 patients, the final diagnosis data was missing. The most common final diagnosis was functional abdominal pain which was found in 200 (21.3%) patients, all of them presented with CAP (Table 4).

## 4. Discussion

This study showed the experience of pediatric endoscopy over a long period of 25 years in the main tertiary hospital in Bahrain. There was a male predominance in pediatric patients who underwent GI endoscopic procedures. This was similar to several published studies from neighboring countries and worldwide (Table 5) [1, 3, 5, 6, 20, 21].

Other studies reported a female predominance [4, 8, 10, 11, 18, 23]. Yet, one study reported an equal male to female ratio [22]. This gender variation might be attributed to cultural and physiological differences.

In the present study, the median age at the time of endoscopic procedure was 8 years. This finding was comparable to other studies [4, 6, 24]. However, several studies reported younger ages (3.2 to 6 years) [2, 16, 22, 25, 26]. This variation at the age of endoscopic procedure might be explained by the discrepancy in the endoscopic indications. For example, in Bawazir et al. study, the mean age was 2.47 years as the only indication was dysphagia in children [13]. However, Akbulut et al. reported a mean age of 12.7 ± 3.4 y, and the only indication was CAP [10].

In this study, the mean number of endoscopic procedures per year was 59 ± 30.9 procedures with a reduction noted after the year of 2011 followed by a marked reduction in the year of 2020. The first reduction can be attributed to the suspension of most of elective endoscopic procedures due to the political unrest related to the 2011 Arab spring. Moreover, the first detected case of coronavirus disease of 2019 (COVID-19) in Bahrain was on the 24 of February 2020, followed by the start of the COVID-19 pandemic. Since then, there was a second reduction in the number of endoscopic procedures up to 73.6%, where also most of the elective procedures have been cancelled or postponed. Although this marked drop in the number of procedures during the exceptional periods (political unstability or COVID-19 pandemic) could not affect the efficacy of the technique of the procedure itself, the indications of the endoscopic procedures might be affected. Only emergency endoscopies were allowed to be performed during that time, while the routine endoscopies were suspended. Accordingly, the diagnostic and therapeutic indications might change as only patients with serious conditions will be considered for endoscopy. A study from the USA by Parasa et al. also reported a significant reduction in total endoscopic procedures during the COVID-19 pandemic (83%) [27]. Similarly, a study from the United Kingdom (UK) by Rutter et al. also showed a reduction ranging from 80 to 95% [28]. He et al. from Australia reported a reduction rate of 66%, which is less compared to our study [29]. Nevertheless, these findings showed the impact of the COVID-19 pandemic on endoscopic services worldwide.

In the current study, the number of UGIE was 3.5 times higher than colonoscopy. Likewise, Sag et al. reported a ratio of 1.94 : 1 between UGIE and colonoscopy [23].

In the present study, most of the endoscopic procedures were diagnostic (89%), while only 11% were therapeutic. Similarly, O'Loughlin et al. reported that 90% of the UGIE were diagnostic [25]. In our study, most of the therapeutic interventions were for esophageal stricture dilatation (7.02%). Yet, in a large multicenter study by Pieczarkowski et al., esophageal stricture dilatation accounted for 3.3% [12].

Endoscopic indications can vary between different countries [4, 16]. The main indication in our study was for CAP (40.9%). This is comparable to what has been reported by several studies [4, 8, 11, 23, 24, 30]. Nonetheless, Ahmad et al. and Ataee et al. reported a higher percentage of CAP of 53% and 97.5%, respectively [11, 30]. This might be attributed to many patients with functional abdominal pain that have been over-investigated with endoscopic procedures [25, 31]. Out of 451 patients presented with CAP in our study, 200 (44.4%) had a final diagnosis as functional abdominal pain, and they had normal endoscopic findings. In fact, we were not satisfied that CAP, which is mostly functional, forms 40.9% of the indications of endoscopic procedures performed for children in our center. Like other studies, this finding can be attributed to the choice of the “line of least resistance” by the treating physician or to the fear of the child's parents/guardians of missinga diagnosis of a life-threating condition like cancer, which is extremely rare in children undertaking UGIE and prompts them to seek unnecessary testing [25, 31]. Yet, Silva et al. looked at this point from the positive side considering performing UGIE as a therapeutic tool besides being a method to exclude other differential diagnosis [31]. They claimed that performing endoscopy in children with CAP can stop the cycle of fear by showing the children and their parents that there is no structural problem causing these pains [31]. We might disagree with this argument. Accordingly, a guideline emphasizes the important indications of endoscopic procedures in children should be developed and implemented to avoid exposing patients to an unnecessary invasive investigation.

UGI bleeding was reported in our study as the second indication of endoscopic procedures (27.4%). Similarly, Faddan et al. and Gadgade et al. studies also reported similar percentages for UGI bleeding (30.1% and 25.3%, respectively) but as the first indication [1, 2]. Despite that, Norsa et al. reported UGI bleeding as the second indication as to our study; but their percentage was higher (48%) [16].

In the present study, the overall yield of endoscopic procedures was 68.1%. The yield differs according to the type of endoscopy performed. Our study showed that the colonoscopy procedure alone gave the best positive yield (82.6%). Likewise, Abdel Azeem et al. reported a positive colonoscopic yield of 85% [22]. Yet, Rabeh et al. and Sağ et al. studies revealed a lower positive colonoscopic yield than what was reported by our study (39% and 55.6%, respectively) [9, 23].

Comparable to our study, Adeniyi et al. reported a positive UGI endoscopic yield of 60.2% [4]. However, UGI procedures in Akbulut et al. and O'Loughlin et al. studies gave lower positive yields of 56.2% and 52%, respectively [10, 25]. The differences in the yield between various studies can be attributed to the differences in the study population [1]. Moreover, the endoscopic yield also differs between the studies based on the different percentages of endoscopic indications. Adeniyi et al. reported a higher yield in patients with UGI bleeding (82.4%), compared to those with CAP (42.9%) and lowest for heartburn/dyspepsia (42.8%) [4]. The biggest impact on the yield was observed in patients with CAP [25]. Despite that CAP was the main endoscopic indication in our study, a finding that has been repeatedly reported by many other studies, our results showed a lower percentage of CAP (40.9%) compared to Adeniyi et al. and Akbulut et al. studies (52.1%, and 65.1%, respectively) [4, 10].

The diagnostic role of endoscopy does not only necessitate the demonstration of organic lesions. Negative yield is also important to reassure parents, to confirm a functional etiology, and to indicate the need for further investigations [1, 31]. The negative endoscopic yield in our study was 31.9%. This is lower than what was reported by O'Loughlin et al. which revealed a negative yield of 48% [25].

## 5. Study Limitations

Like other retrospective studies, this study was limited by missing some data related to patients' demography and the endoscopic yields. Despite that Salmaniya Medical Complex is the main center that offers pediatric gastroenterology endoscopy services in the country; this study is a single-center study; and generalization to the whole pediatric population of Bahrain might be inappropriate. Furthermore, not all the patients had their final diagnosis available as the collection of laboratory, radiological, and histopathological results was not the aim of this study. Missing data of histological biopsy results makes the comparison between the endoscopic and histological findings difficult to achieve. Silva et al. study showed that histopathological yield (69.6%) exceeds the overall macroscopic endoscopic findings (51.3%) [31]. Despite these limitations, this study is the first work tackling endoscopic procedures in children from Bahrain with a relatively large sample size. Findings of this study are very important for any physician dealing with pediatric age groups, and it will have a reflection on their clinical practice as new lines of action and efficiencies can be derived.

## 6. Conclusions

Findings of this study underline the importance of endoscopic procedures, especially colonoscopy, in diagnosis and treatment of pediatric GI diseases. They also emphasize the importance of careful selection of the type of endoscopic techniques used based on the patients' symptoms and the expected endoscopic yield. In patients with less specific symptoms such as those presented with CAP, functional origin should be kept in mind, and endoscopy should be reserved as a second-line tool, after failure of all other investigations to find the cause of child's symptoms. This is to avoid unnecessary use of expensive and invasive procedures. Accordingly, a proposal of guidelines to investigate patients before endoscopy will be of value considering the existing limitations to access to endoscopic procedures for those in need.

## Figures and Tables

**Table 1:** Demographic data of pediatric patients who underwent gastrointestinal endoscopy (*n* = 1,101).

Demographic data	Patient's number (%)
Sex (*n* = 1098)	
Male	589 (53.6)
Female	509 (46.4)
Nationality (*n* = 1088)	
Bahraini	1029 (94.6)
Non-Bahraini	59 (5.4)
Age (y) (*n* = 1087)	
Mean ± SD	7.1 ± 4.4
Median (IQR)	8.0 (3, 11)
Age group (y) (*n* = 1087)	
0-4.9	371 (34.1)
5-9.9	326 (29.9)
10-14.9	370 (34.0)
15-18	20 (1.8)
Type of endoscopic procedure performed (*n* = 1101)	
UGIE alone	833 (75.6)
Colonoscopy alone	80 (7.3)
Both procedures combined	188 (17.0)

SD: standard deviation; IQR: interquartile range; UGIE: upper gastrointestinal endoscopy. Values are presented as number (%), mean ± SD or median (IQR).

**Table 2:** Indications of gastrointestinal endoscopic procedures in children (*n* = 1,101).

Indications of endoscopy	Patients' *n* (%)∗
Chronic abdominal pain	451 (40.9)
Vomiting and hemorrhage	302 (27.4)
Chronic diarrhea	92 (8.4)
Lower gastrointestinal bleeding	90 (8.2)
Chronic reflux disease without improvement with medications	64 (5.8)
Impaired growth	62 (5.6)
Foreign body ingestion	59 (5.4)
Dysphagia	56 (5.1)
Constipation	50 (4.5)
Acid base ingestion	33 (2.9)
To rule out celiac disease	32 (2.9)
To rule out inflammatory bowel disease	30 (2.7)
Dilatation of strictures	26 (2.4)
Abdominal distension	22 (1.9)
Reassessment of known disease †	19 (1.7)
Insertion/replacement of feeding tube	5.0 (0.5)
Perianal lesions (mass, fistula)	5.0 (0.5)
Miscellaneous^‡^	12 (1.1)

∗ Values are presented as number (%). Patients may have more than one endoscopic indication. ^†^One for Crohn's disease, two for eosinophilic esophagitis and 16 for abdominal symptoms of cystic fibrosis. ^‡^Cyanosis, anorexia, lower limb edema, obstructive jaundice, and ingestion of boiling water to rule out colonic duplication, intestinal lymphangiectasia, and intestinal polyps each had one procedure; feeding intolerance and pyloric stenosis each had two procedures.

**Table 3:** Endoscopic yield in children underwent gastrointestinal endoscopic sessions (*n* = 1,296).

Type of procedure	Total procedure	Total yield	Endoscopic yield =1052 (81.2)		*P* value∗
			Positive =716 (68)	Negative =336 (32)	
UGIE only	955 (73.6)	819 (85.8)	537 (65.6)	282 (34.4)	0.001
Colonoscopy only	103 (7.9)	46 (44.7)	38 (82.6)	8.0 (17.4)	0.030
Combined procedures	238 (18.3)	187 (78.6)	141 (75.4)	46 (24.6)	0.018

UGIE: upper gastrointestinal endoscopy. Values presented as number (%). ^∗^Fisher's exact test. *P* value <0.05 is statistically significant.

**Table 4:** Final diagnosis of children who underwent gastrointestinal endoscopy (*n* = 941).

Final diagnosis	Number (%)
Functional abdominal pain	200 (21.3)
Nonspecific gastritis	145 (15.4)
*Helicobacter pylori* gastritis	131 (13.9)
Inflammatory bowel disease∗	109 (11.6)
Celiac disease	86 (9.1)
Gastroesophageal reflux disease	69 (7.3)
Foreign body ingestion	61 (6.5)
Esophageal stricture †	46 (4.9)
Mallory Weiss syndrome	44 (4.7)
Eosinophilic esophagitis	27 (2.9)
Duodenal ulcer	24 (2.6)
Esophageal ulcers	16 (1.7)
Peptic ulcer disease	14 (1.5)
Cow's milk protein allergy	12 (1.3)
Anal fissure	12 (1.3)
Intestinal polyposis ‡	8 (0.9)
Chronic liver disease with esophageal varices	8 (0.9)
Caustic ingestion	8 (0.9)
Rectal ulcers	8 (0.9)
Eosinophilic gastroenteritis	7 (0.7)
Hiatal hernia	7 (0.7)
Non-Hodgkin lymphoma	6 (0.6)
Lymphoid nodular hyperplasia	4 (0.4)
Hirschsprung disease	3 (0.3)
Insulin dependent diabetes mellitus	3 (0.3)
Cystic fibrosis	3 (0.3)
Nonorganic failure to thrive	3 (0.3)
Intestinal lymphangiectasia	3 (0.3)
Acute pancreatitis	3 (0.3)
Cyclic vomiting	2 (0.2)
Esophageal necrosis and perforation	2 (0.2)
Viral enteritis	2 (0.2)
Meckel's diverticulum	2 (0.2)
Others§	6 (0.6)

Values are presented as number (%). Patients may have more than one final diagnosis (*n* = 233). ∗Ulcerative colitis (*n* = 58) and Crohn's disease (*n* = 51), †esophageal stricture posttracheoesophageal fistula repair (*n* = 35), ‡juvenile polyps (*n* = 7) and one patient with Cowden syndrome polyps, §postjejunal atresia repair tube insertion, Barret esophagus, gastric outlet malrotation, duodenal atresia, angiodysplasia, hyperelimination postgastroschisis repair; each in one patient.

**Table 5:** Summary of studies regarding endoscopies from neighboring countries and worldwide.

Country	Author, year	Duration	Patients number	Age (y)	Sex ratio	Most common indication			Yield
						1st indication (%)	2nd indication (%)	3rd indication (%)	
Bahrain	Isa and Alfayez^*^, 2022	1995-2020	1101	8.0 ± 8.0	M > F	CAP (40.9)	Vomiting and hemorrhage (27.4)	LGIB (8.2)	68.1%
KSA	Saeed et al. [3]	2010-2015	37	9.6 ± 2.3	M > F	Dysphagia (56.7)	Vomiting (48.6)	Food bolus impaction (21.6)	NR
Iran	Mirrahimi et al. [20]	2019	43	4.98 ± 3.8	M > F	Hematemesis	Melena	Coffee grounded vomiting	NR
Egypt	Abd Allah et al. [6]	2014-2015	40	7.8 ± 3.1	M > F	Bleeding per rectum (42.5)	CAP (25)	Vomiting, hematemesis and melena (15)	80%
Egypt	Faddan et al. [1]	2004-2013	177	<6 m	M > F	UGIE: Hematemesis (30) Colonoscopy: rectal bleeding (19.4)	UGIE: Vomiting (18.4) Colonoscopy: bloody diarrhea (13.6)	UGIE: Melena (5.8)	70.6%
Egypt	Abdel Azeem et al. [22]	2016-2017	40	5.7 ± 2.8	M = F	Bleeding per rectum (80)	CAP (10)	Weight loss (5)	85%†
Tunisia	Rabeh et al. [9]	2010-2017	210	Children	NR	LGIB (57.1)	IBD (29)	NR	39% †
Turkey	Sağ et al. [23]	2010-2017	39	8.2 ± 4.0	F > M	UGIE: CAP (65.7) Colonoscopy: abdominal pain (38.9)	UGIE: dyspepsia (17.1) Colonoscopy: chronic diarrhea (22.2)	Chronic diarrhea (8.6)	55.6%†
France	Norsa et al. [16]	2017	237	3.2	NR	Foreign body ingestion (24)	GI bleeding (48)	Caustic ingestion (63)	NR
Spain	Flores-González et al. [21]	2016-2017	88	Median 7	M > F	Celiac disease (33)	Epigastric pain (13)	Esophagitis (8)	NR
Australia	Singh et al. [5]	2011-2015	652	13 (0.4-18)	M > F	IBD (57.9)	Rectal bleeding (10)	CAP (10)	NR
USA	Pant et al. [24]	NR	437283	0-19	NR	LGIB (30.2)	UGIB (20.3)	Unspecified GI bleeding (10.4)	NR

∗ The present study; †colonoscopy yield. CAP: chronic abdominal pain; LGIB: lower gastrointestinal bleeding; NR: no records; UGIE: upper gastrointestinal endoscopy; IBD: inflammatory bowel disease; USA: United States of America; UGIB: upper GI bleeding.

## Data Availability

The datasets generated during and/or analyzed during the current study are available from the corresponding author on reasonable request.
